# Supplementary figures and images for: Minimal transcriptional regulation of horizontally transferred photosynthesis genes in phototrophic bacterium Gemmatimonas phototrophica
Source: mSystems. 2024 Aug 27;9(9):e00706-24. doi: 10.1128/msystems.00706-24 (PMC11406998; doi:10.1128/msystems.00706-24)

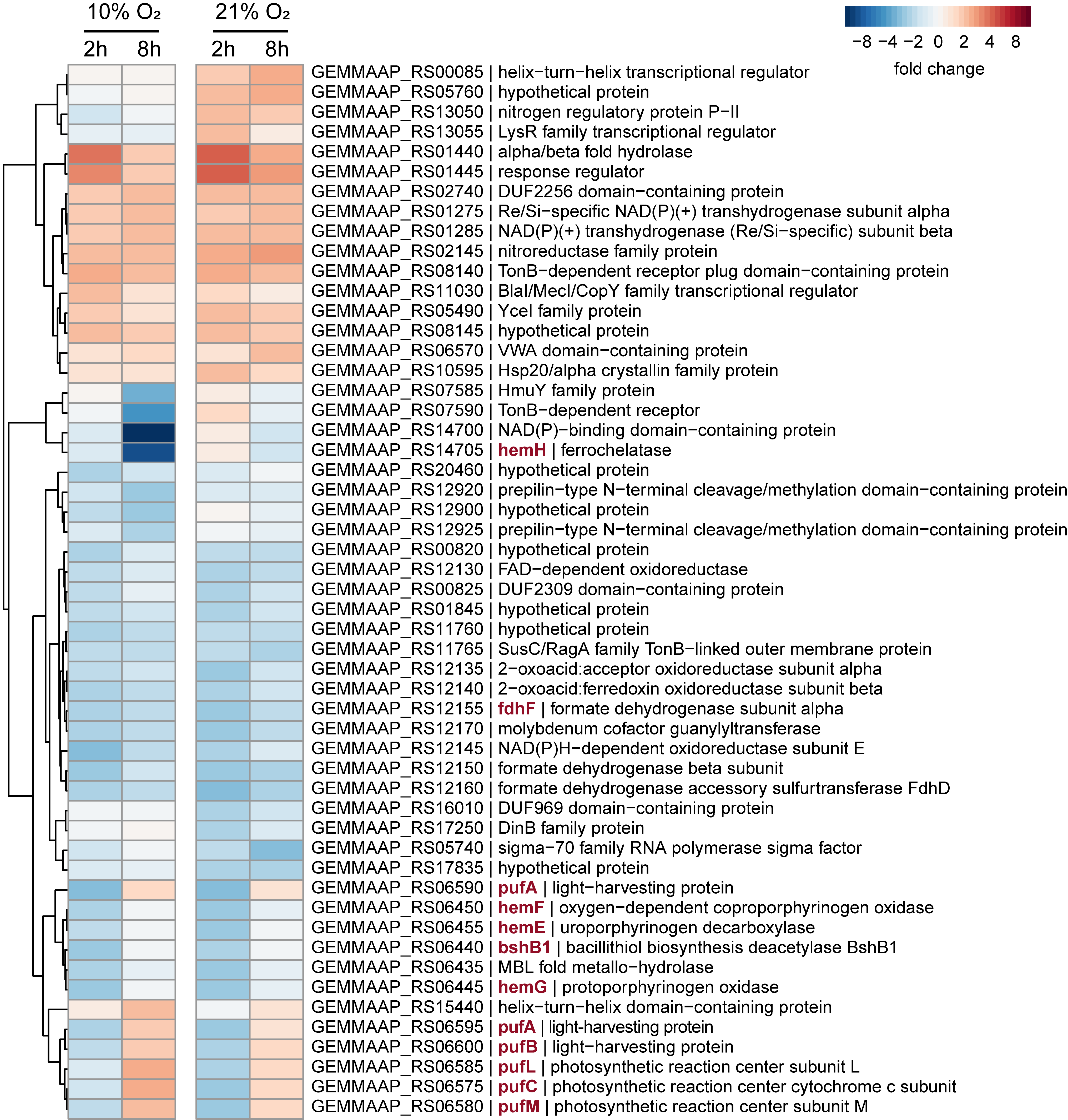

Supplement: Figure S1 — Transcriptional response to light under different O2 concentrations. [file msystems.00706-24-s0001.tif]

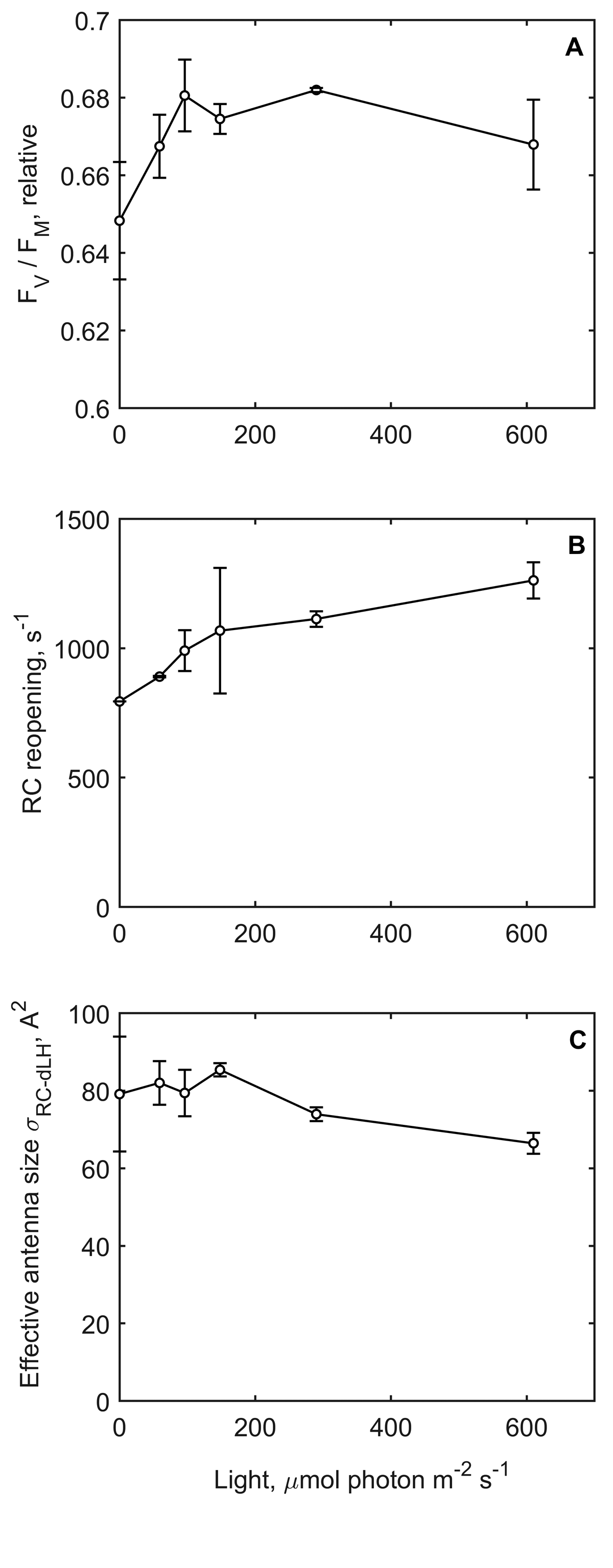

Supplement: Figure S2 — The effect of light intensity on the activity of reaction center with two rings of light-harvesting antennae. [file msystems.00706-24-s0002.tif]
