# Supplementary material for: Minimal transcriptional regulation of horizontally transferred photosynthesis genes in phototrophic bacterium Gemmatimonas phototrophica
Source: mSystems. 2024 Aug 27;9(9):e00706-24. doi: 10.1128/msystems.00706-24 (PMC11406998; doi:10.1128/msystems.00706-24)
Supplement: Legends — for Figures S1 and S2. [file msystems.00706-24-s0004.docx]

**Supplementary Figure Legends**

**Supplementary Figure S1 – Transcriptional response to light under different O_2_ concentrations.** Heatmap showing the fold changes after 2 and 8 h in light compared to the dark control for both O_2_ concentrations separately. Selected genes, in particular from the PGC, are highlighted.

**Supplementary Figure S2 – The** **effect of light intensity on the activity of reaction center with two rings of light-harvesting antennae.** The yield of primary photochemical reactions, F_V_/F_M_ (A), the reopening rate of the reaction center with two rings of light-harvesting antennae, *k_re-open_* (B), and the functional antenna cross section, σ_RC-dLH_ (C) responded to the increasing light intensities during growth of *G*. *phototrophica* AP64^T^ cells.
